# Some considerations in optimizing the Medical Physics Match

**DOI:** 10.1002/acm2.12732

**Published:** 2019-10-21

**Authors:** Richard V. Butler, John H. Huston, George Starkschall

**Affiliations:** ^1^ Department of Economics Trinity University San Antonio TX USA; ^2^ Department of Radiation Physics The University of Texas MD Anderson Cancer Center Houston TX USA

For several years, many medical physics educational programs in the United States and Canada have been participating in a residency application matching program (MedPhys Match) in assigning graduates of CAMPEP‐accredited graduate programs to residency programs. Patterned after the National Resident Matching Program (The Match) for medical school graduates, the MedPhys Match is designed to maximize the satisfaction of the residency selection process for both the residency candidate and the residency program.

Originally developed by Roth,^1^ the match algorithm requires the residency candidate to rank order residency programs and the residency programs to rank order residency candidates. The match algorithm then tries to combine the highest ranking candidate with highest ranking program. In 1962, Gale and Shapley demonstrated that this algorithm will always provide a stable solution.^2^ In 2012, Roth and Shapley were awarded the Nobel Prize in Economics for the matching algorithm.

Several problems have arisen in the application of this algorithm to the MedPhys Match. One problem is that the number of residents entering any given program is small. In 2018, a mean of 1.4 applicants per program began residency training.^3^ A second problem is the imbalance between the number of graduates applying to residency programs and the number of residency positions. In 2018, 79% of graduates of CAMPEP‐accredited graduate programs were accepted into residency programs.^4^ Consequently, to ensure a match, candidates interview at many programs. There is also a harmful feedback mechanism here. As applicants apply to more programs, the acceptance rate at each program declines. Consequently, applicants may apply to even more programs to increase their perceived probability of acceptance into a program. This is costly for the candidates in terms of travel expenses, and costly for the interviewing faculty in terms of time away from research, clinic, and teaching.

The problem, then, that must be solved is to determine the optimal number of programs to which a prospective residency candidate should apply. On one hand, applying to very few programs is desirable because of the lower travel cost, but undesirable because of the lower probability of being matched. On the other hand, applying to a large number of programs is undesirable because of the increased cost, but desirable because of the higher probability of being matched. The solution to this problem is not easy to find since the solution is likely to be candidate‐specific. The purpose of this paper is to present some factors that need to be taken into account in achieving a solution.

Fundamentally, finding an applicant's optimal number of applications is a straightforward constrained maximization problem, in which the applicant compares the benefits and costs of additional applications subject to a budget constraint. The search should stop when the marginal benefit of an additional application equals the marginal cost (since we would expect the marginal benefit to be declining and the marginal cost rising as a function of the number of applications). Presumably applicants would have a pretty decent sense of what their cost function looks like; the challenge would be in quantifying the benefit function since it is probabilistic. The marginal benefit of applying to the applicant's nth program is a function of the probability of getting a match with that program. As n rises, it is more and more likely that a match would already been achieved with a higher ranked program and thus the probability of a match with the nth program falls. The proof of this is as follows:

Imagine a list of possible residencies with the first being the student's favorite and the last being his/her least favorite. *p_i_* is the probability of matching the ith residency on a student's list given that the student did not match with any his/her *i‐1* other possible residencies. So for the second residency, the probability of a match, P_i_, is the probability of not matching with the first residency times the probability of matching the second P_2_ = (1 − *p*_1_)*p*_2_. For a student considering interviewing with the ith residency on the list the probability of matching is:

$$P_{i}=\left(1-p_{1}\right)\left(1-p_{2}\right)\left(1-p_{3}\right)\ldots \ldots \left(1-p_{i-1}\right)p_{i}$$

Each of those terms is a fraction less than one, so as *i* rises, the probability of matching falls. Since the student's list is organized in order of preference, the benefit of the ith position is also falls as i increases. So the expected marginal benefit P_i_B_i_ declines as the student interviews with additional residencies.

In recent times, the advent of electronic applications and online job‐matching platforms has lowered the marginal cost of applying almost to zero. This has led to huge increases in the number of applicants that hiring universities must review. For example, in 2005 a faculty vacancy in the Department of Economics at Trinity University attracted 64 applicants for that position. Last year the Department had 536 candidates apply. Because it is impossible for a hiring committee to give careful consideration to that many applicants, institutions are looking for ways to reduce the flood by, in effect, raising the marginal cost of applying. This can be accomplished by, for example, requiring candidates to provide answers to questions unique to that position. That seems to have helped a little. However, if the number of medical physics graduates seeking residencies is small, this may not be a useful solution.

Because the problem of optimal applications is an economics problem, there has been a search for solutions and a developing literature on the subject. Balter et al.^5^ show that limiting the number of applications candidates can submit is superior to limiting the number of applications a program can evaluate. Entering an application limit into the Gale/Shapley algorithm that underlies the matching process, the authors conclude that "the optimal limit in the number of applications balances the tradeoff between being unmatched and gaining a better match in the aggregate, and the benefit can be considerable if the graduates' preferences over the positions are not very correlated." In other words, limiting the number of applications can actually result in better outcomes for the applicants as well as the lower costs for the institutions. One way to possibly identify that limit is to take a sample of a few years' residency markets and determine how far down their list the lower‐ranked candidates had to go to get a match. Presumably, interviews beyond that point are very likely to have negative net benefits.

Another approach to a solution is "signaling." A program would be permitted to notify a small number (somewhere between three and five) of applicants prior to interviews that it is seriously interested in them. This gives the applicant useful information about his/her chances at that particular program and so makes the benefit function a bit less fuzzy. Because the problem in medical physics seems to be more at the interview stage than the initial application stage, some form of signaling by institutions offering residencies might help reduce uncertainty so that at least some applicants could focus on the places where they have good chance and pass on visits to some of their more marginal options.

In conclusion, the problem of optimizing the number of residency programs to which a medical physics graduate should apply is a cost‐benefit problem. The incremental cost of applying to an additional program is essentially the cost of travel to that program for an interview; the benefit has yet to be quantified. Strategies for mitigating the cost of a large number of applications include limiting the number of programs to which a candidate may apply and/or allowing programs to notify candidates if they are seriously interested in them.

## References

[1] Roth AE , Peranson E . The redesign of the matching market for American physicians: some engineering aspects of economic design. Am Econ Rev. 1999;89(4):748–780. 29115787 10.1257/aer.89.4.748

[2] Gale D , Shapley LS . College admissions and the stability of marriage. Am Math Mon. 1962;69:9–15.

[3] Dogan N . "CAMPEP Residency Program Report." Presented at the AAPM annual meeting. San Antonio, TX, 2019, July 16.

[4] Clark BG . 2019 "CAMPEP Graduate Program Report." Presented at the AAPM annual meeting. San Antonio, TX, July 16.

[5] Balter J , Rancan M , Senyuta O . Limit your applications. Dealing with congested markets in the matching procedure. Int J Comput Econ Econometrics. 2016;6(4):413–431.

